# Recognition of immunogenomic signature and prognostic value of the subtype of epithelial-mesenchymal transition in breast cancer

**DOI:** 10.1016/j.bbrep.2026.102456

**Published:** 2026-01-19

**Authors:** Wei Liang, Zi-ying Wang, Quan-feng Shao, Yuan-yuan Li, Bei Zhu, Xi-hu Qin, Wei-xian Chen

**Affiliations:** a Dalian Medical University, Dalian, Liaoning Province, 116000, China; b Department of Breast Surgery, Changzhou No.2 People's Hospital, The Third Affiliated Hospital of Nanjing Medical University, Changzhou, Jiangsu Province, 213000, China; c Post-doctoral Working Station, Changzhou No.2 People's Hospital, The Third Affiliated Hospital of Nanjing Medical University, Changzhou, Jiangsu Province, 213000, China

**Keywords:** Epithelial-mesenchymal transition, Immunology, Genomics, Bioinformatics, Breast cancer

## Abstract

**Background:**

Accumulating evidence has revealed that epithelial-mesenchymal transition (EMT) plays a crucial role in tumor progression and the immune microenvironment, which further results in a high rate of recurrence and metastasis. The EMT immune signaling pathway provides a great perspective for designing personalized therapies.

**Methods:**

In this study, 1223 RNA-seq samples were obtained from the TCGA-BRCA dataset. A total of 381 EMT-related differentially expressed genes were analyzed and combined with clinical parameters, and the matrix was randomly divided into training and testing cohorts at a ratio of 7:3. The training cohort was used to develop an EMT signature, including *GKN2*, *FZD2*, *NDRG2*, *SCUBE2*, *ALX4*, *CCL19*, *SDC1*, *EZR*, *CPEB1*, and *HRG* genes, and the accuracy of this signature was validated by testing and GSE158309 cohorts.

**Results:**

A risk score model and clinical parameters were used to establish a nomogram for predicting prognosis. The C-index (0.719), calibration curves, and model comparison with four previous studies demonstrated the reliability of the EMT signature, the biological phenotypes of which were tested for functional enrichment, immune infiltration, and tumor mutation. Additionally, patients' responses to immunotherapy and chemotherapy were assessed. Our results showed that the low-risk group had higher immune infiltration, tumor mutational burden, microsatellite instability levels, immune checkpoint inhibitor expression, tumor immune dysfunction and exclusion scores, and immunophenoscore, which could predict patient sensitivity to immunotherapy. Moreover, low-risk patients exhibit better sensitivity to chemotherapy.

**Conclusion:**

This novel EMT signature offers excellent potential for predicting the prognosis, tumor immune heterogeneity, and therapeutic responses in breast cancer.

## Highlights

• We identified a novel EMT signature to predict prognosis of breast cancer patients.
• We demonstrated the reliability of the EMT signature by several ways.
• The low-risk patients exhibit better sensitivity to chemotherapy.

## 1 Introduction

Despite advanced treatment, recurrence and metastasis are the leading causes of breast cancer (BRCA) mortality worldwide [1]. Once local recurrence and distant colonization occur, the lesion is difficult to eradicate and the risk of drug resistance increases, eventually leading to a lower survival rate and worse prognosis [2]. Recently, an increasing number of researchers have taken a hard look at tumor biology. Epithelial-mesenchymal transition (EMT) is an evolutionarily conserved developmental process implicated in carcinogenesis and metastasis of cancer cells by promoting mobility, invasion, immune evasion, and resistance to apoptosis [3].

EMT has been considered to be a critical hallmark of tumorigenesis and cancer progression. Specifically, neatly arranged epithelial cells would lose their apical-basal polarity, cell-cell junctions, and adherence to the basement membrane to obtain mesenchymal cells with a shuttle shape that enables migration and invasion, always accompanied by the alteration of specific biomarkers (proteins such as E-cadherin, N-cadherin, vimentin, and β-catenin, and factors such as *Snail, Slug, Twist, and Zeb*) [4]. The morphological evolution of the EMT enables further biotransformation and signal transduction. Significantly, mesenchymal-epithelial transition is the reverse of this process, which is believed to trigger the cessation of migration, inducing cancer cells to proliferate and seed into the target organ [5]. The plasticity and extension of EMT have increased research difficulty; however, targeting the EMT pathways remains an attractive strategy for cancer treatment. Cancer immunotherapy has recently achieved remarkable success as an emerging treatment. Immune cells in the tumor microenvironment exhibit excellent tumor-promoting and tumor-suppressing activities [6]. The abundance and intensity of these immune cells are heterogeneous, resulting in differential immunotherapy responses and clinical outcomes [7]. Studies have indicated that EMT plays a pivotal role in tumor immunosuppression and immune evasion [8]. In turn, immune cell recruitment strengthens the ability of tumor cells to undergo EMT [9].

However, existing EMT-derived prognostic signatures largely emphasize metastatic potential. A critical gap remains in our understanding of how EMT modulates the heterogeneous tumor immune microenvironment and influences therapy response, limiting the development of precise treatments. To address this gap, this study aims to develop and validate a novel EMT gene signature intrinsically linked to immune regulation. We hypothesize that such a signature will provide robust prognostic stratification and identify patient subsets with distinct immune phenotypes and differential therapeutic susceptibilities, offering a new tool for precision oncology in breast cancer.

## 2 Methods and materials

### 2.1 Data collection and preprocessing

Tumor mutational burden (TMB) data, RNA-Seq data, and clinical parameters of BRCA samples were downloaded from The Cancer Genome Atlas (TCGA) (https://portal.gdc.cancer.gov/), including 1223 samples. GSE158309, whose RNA-seq matrix and clinical pathology parameters were used for external validation, was downloaded from the Gene Expression Omnibus (GEO) database (https://www.ncbi.nlm.nih.gov/geo/). All gene probes were transformed into gene symbols using the public database, Ensembl (http://uswest.ensembl.org/index.html). These gene matric |log FC|>1 were de-duplicated and standardized using log2(rt+1). Genes whose normalized expression was zero in over half of the samples or whose expression was constant or invariable were excluded. Approximately 1184 EMT-related genes were collected from an accessible database (dbEMT2.0: http://dbemt.bioinfo-minzhao.org/). Ethical approval by an ethics committee was not requested, considering that the BRCA patient information from public databases was open-access.

### 2.2 Differential expression analysis of genes

The R programming statistical package “edgeR” was used to analyze the differentially expressed genes between the tumor and adjacent samples, which were intersected with 1184 EMT-related genes using a Venn plot, and 381 differentially expressed EMT-associated genes (EAGs) were identified for subsequent analyses.

### 2.3 Immune infiltration analysis

Diverse computational deconvolution methods have been adapted to purify the proportions of various immune cells in heterogeneous samples [10]. In the present study, we selected several algorithms to quantify the abundance of immune cells and compared the differences between the high- and low-risk groups, including the ESTIMATE, CIBERSORT, and MCP-counter methods. The immune, stromal, and ESTIMATE scores of each patient were determined using the ESTIMATE algorithm [11]. We then used CIBERSORT to assess the proportion of 22 types of immune cells in BRCA tissues, mainly covering several types of B cells, plasma cells, T cells, NK cells, monocytes, macrophages, mast cells, eosinophils, and neutrophils [12]. Finally, the MCP-counter method evaluates the quantity of 10 immune cells in each sample, including T cells, cytotoxic lymphocytes, B lineages, NK cells, monocytic lineages, myeloid dendritic cells, neutrophils, endothelial cells, and fibroblasts [13]. We also conducted Spearman's correlation analysis between the risk score and the 10 immune cells from the MCP-counter analysis using the “GGally” R package.

### 2.4 Gene set enrichment analysis

To evaluate the correlation between EMT signature and immune infiltration, to explore the potential mechanism in-depth, we performed gene set enrichment analysis through the Bioconductor package “clusterProfiler” to identify the specific functional enrichment pathway between high-risk and low-risk groups. The gene sets used in this study included the Kyoto Encyclopedia of Genes and Genomes (KEGG) and Genome Ontology (GO).

### 2.5 Sensitivity prediction to therapies in patients with different EMT risk scores

More biomarkers are required to predict drug sensitivity considering the rapid development of precise therapies for advanced BRCA. We used the EMT signature to guide clinical treatment. First, the “ggstatsplot” R package was used to test the relationship between risk scores and typical immune checkpoint inhibitors (ICIs), such as *PDCD1, CD274, CTLA4, PDCD1LG2, LAG3, and HAVCR2*. The patients’ potential response to immunotherapy was also determined using the tumor immune dysfunction and exclusion (TIDE) and immunophenoscore (IPS). Generally, the lower the TIDE score and IPS, the better is the prognosis of patients with tumors. Taking advantage of the “pRRophetic” package [14,15], we investigated the predictive capacity of the EMT signature in response to 138 chemotherapeutic drugs. Subsequently, the values were transformed normally.

### 2.6 Immunohistochemical validation in human protein atlas database

The protein expression levels of EMT signature genes are displayed in the Human Protein Atlas (HPA) database (https://www.proteinatlas.org/), which covers the distribution and expression information of over 24000 proteins in normal human and tumor tissues. Immunohistochemical staining images were downloaded for visualization, and antibody staining intensity comparisons were used to analyze differences in protein expression between normal breast and BRCA tissues.

### 2.7 Statistical analysis

The log-rank *t*-test, Wilcoxon rank-sum test, and Kaplan-Meier survival curve were used to calculate the statistical significance between the high-risk and low-risk groups. Based on clinicopathological parameters (age, sex, clinical tumor stage, clinical lymph node stage, and distant metastasis status), we employed univariate and multivariate Cox analyses to identify independent prognostic factors. LASSO-Cox regression analysis was performed to remove the overfitted genes using the basic function of the “coxph” R package. Time-dependent receiver operating characteristic (ROC) curves at 1, 3, and 5 years were generated, and the area under the ROC curve (AUC) was visualized to assess the accuracy using the R package “survivalROC.” A nomogram for prognostic prediction was implemented using the R package “rms.” Relationships between cell-to-cell and gene-to-cell were computed using the “Spearman” method, and a correlation test was used to determine their respective significance. All statistical analyses were performed using R 4.1.1 version.

## 3 Results

### 3.1 Recognition of the differentially expressed EMT-associated genes (EAGs)

**Fig. 1:**
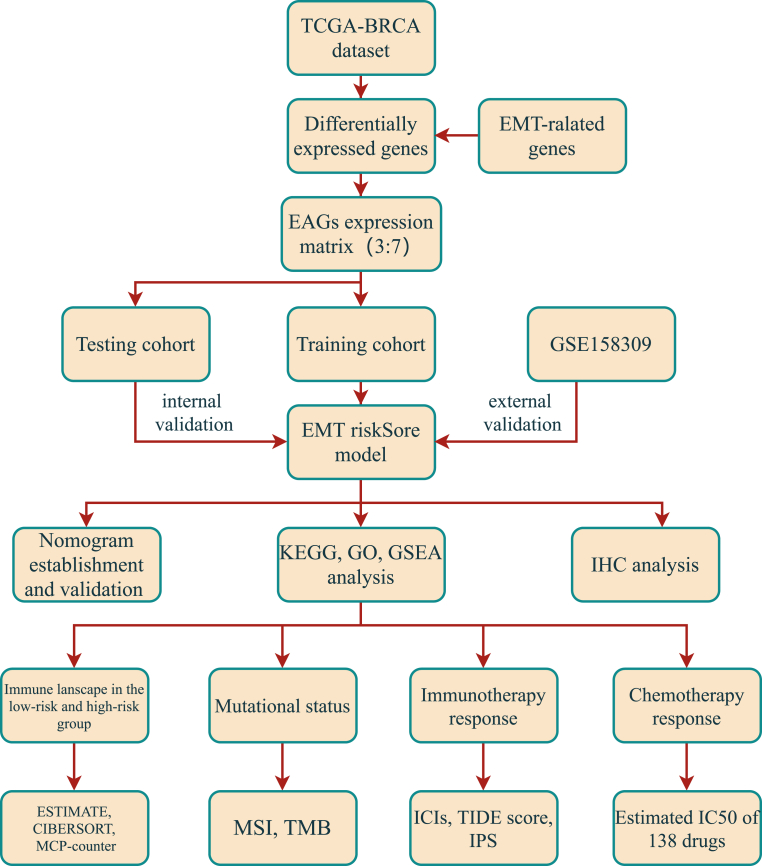
Flowchart of the present study.

The transcriptome expression matrix for BRCA was obtained from TCGA database, which included 1110 tumor samples and 113 adjacent samples. A total of 8130 differentially expressed genes were identified (log |FC|>1, *adj P < 0.05*) after annotation and normalization, with 2520 down-regulated and 5610 up-regulated (Supplementary Fig. 1A). A Venn diagram intersecting 1184 EAGs and 381 overlapping genes between the two datasets was acquired (Supplementary Fig. 1B). The following analyses and workflow were based on the 381 EAGs matrix (Fig. 1).

### 3.2 Development and evaluation of EMT signature for prognosis prediction

**Fig. 2:**
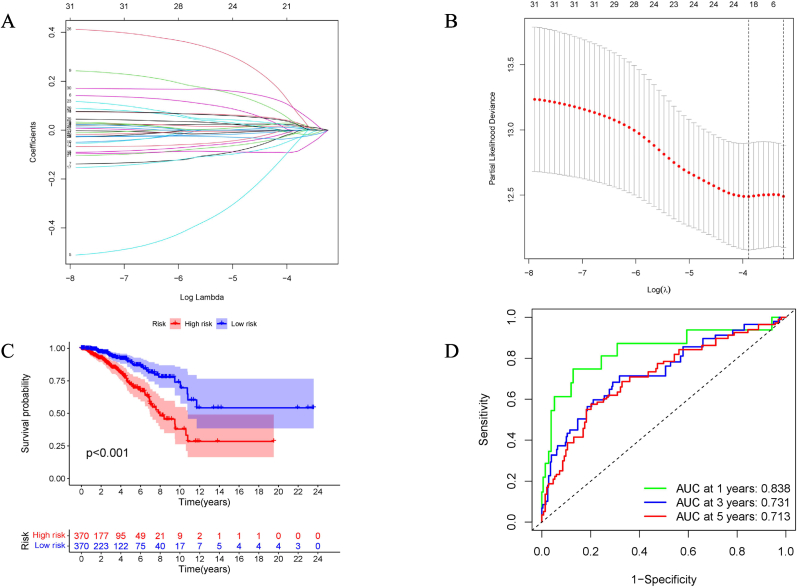
**Statistical analysis for constructing EMT signature using the TCGA training cohort.** (A) LASSO-Cox regression coefficient profiles of 31 survival-related genes. Lower horizontal axis showed the Log(lambda); upper horizontal axis represents the total number of variables. Left longitudinal axis represents the value of coefficients. (B) Accordion diagram was visualized to cross-validate the LASSO-Cox regression model for tuning parameter choosing. Horizontal axis represents the Log(lambda), while longitudinal axis represents partial likelihood deviance. The first black dotted line indicated the optimal lambda value. (C) Kaplan-Meier curve indicated the survival difference between high-risk and low-risk groups (*P < 0.001*). (D) AUCs at 1, 3, and 5-year of ROC curves.

To search for potential biomarkers for prognosis prediction among these 381 EAGs, a backward variable selection procedure, univariate Cox, LASSO-Cox, and multivariate Cox regression were adapted to the expression data and clinical information of 1058 patients in the TCGA-BRCA dataset. These samples were randomly divided into training and testing cohorts in a ratio of 7:3, to ensure reproducibility, the split was performed with a fixed random seed of 123. Given the imbalanced nature of our classification labels, we employed stratified sampling to preserve the original class distribution in both sets. Training cohort was used for recognition of a new signature. Univariate Cox regression (*P < 0.05*) screened out 31 genes related to overall survival (OS, Supplementary Table 1). LASSO-Cox regression with 10-fold cross-validation was applied to delete the overfitted genes (Fig. 2A–B), quantifying the independent prognostic genes, the final signature was entitled by “EMT signature”, which composed of ten genes (*GKN2*, *FZD2*, *NDRG2*, *SCUBE2*, *ALX4*, *CCL19*, *SDC1*, *EZR*, *CPEB1*, and *HRG*). The coefficients of these genes were weighted using multivariate Cox regression and then transformed into a risk score as follows: $\text{risk}\;\text{score}=\sum_{n=1}^{a}\left({\text{Coef}}_{n}*x_{n}\right)$. *Coefn* is the coefficient and *Xn* represents the expression level of the corresponding gene in each sample.

Univariate and multivariate Cox regression analyses with clinical parameters (Age, Sex, and TNM stage) showed independent predictive power for the EMT signature. Moreover, we extracted the clinical information (Age, Sex, TNM stage, T stage, N stage, and M stage) of the training and testing groups for differential analysis using the log-rank *t*-test, with a p-value for each variable >0.05, indicating that there was no clinical deviation in the random grouping (Table 1). The EMT signature score was estimated for each patient in the training cohort. Subsequently, 740 patients were stratified into high- and low-risk groups using the median value (0.8) as the risk cut-off. The low-risk group had a significantly higher OS than the high-risk group (*P < 0.001*), with respective survival rate of 0.89 and 0.74 in 5 years (Fig. 2C). The AUCs of the time-dependent curves for 0.838, 0.731, and 0.713 at 1, 3, and 5-year OS (Fig. 2D).

**Table 1:** Differential analysis of the clinical parameters between train and test cohort.

Covariates	Type	Total	Test	Train	Pvalue		
Age	≤65	751(71.12 %)	230(72.78 %)	521(70.41 %)	0.4795		
	>65	305(28.88 %)	86(27.22 %)	219(29.59 %)			
Sex	Female	1044(98.86 %)	313(99.05 %)	731(98.78 %)	0.954		
	Male	12(1.14 %)	3(0.95 %)	9(1.22 %)			
TNM	stageI	178(16.86 %)	58(18.35 %)	120(16.22 %)	0.3134		
	stageII	615(58.24 %)	186(58.86 %)	429(57.97 %)			
	stageIII	246(23.3 %)	70(22.15 %)	176(23.78 %)			
	stageIV	17(1.61 %)	2(0.63 %)	15(2.03 %)			
T_Stage	T1	268(25.38 %)	75(23.73 %)	193(26.08 %)	0.5728		
	T2	618(58.52 %)	183(57.91 %)	435(58.78 %)			
	T3	136(12.88 %)	47(14.87 %)	89(12.03 %)			
	T4	34(3.22 %)	11(3.48 %)	23(3.11 %)			
N_Stage	N0	511(48.39 %)	160(50.63 %)	351(47.43 %)	0.2298		
	N1	355(33.62 %)	111(35.13 %)	244(32.97 %)			
	N2	117(11.08 %)	28(8.86 %)	89(12.03 %)			
	N3	73(6.91 %)	17(5.38 %)	56(7.57 %)			
M_Stage	M0	892(84.47 %)	273(86.39 %)	619(83.65 %)	0.2059		
	M1	17(1.61 %)	2(0.63 %)	15(2.03 %)			
	unknown	147(13.92 %)	41(12.97 %)	106(14.32 %)			

**Fig. 3:**
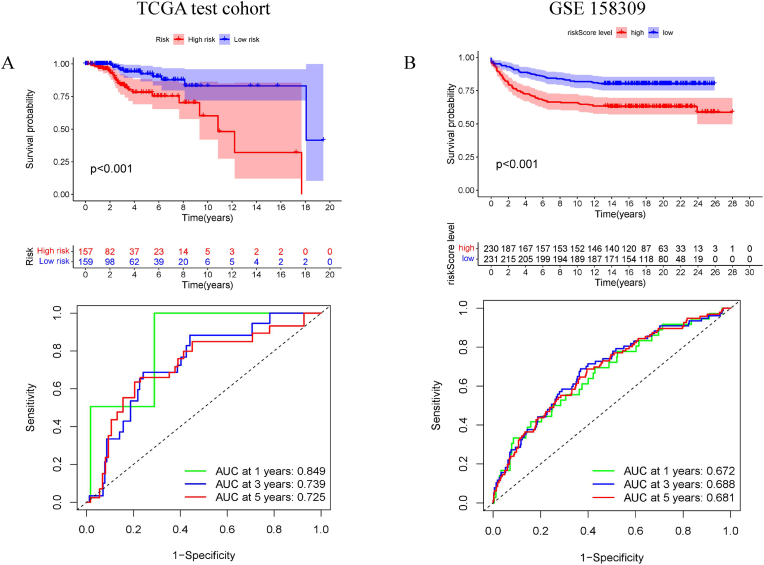
**Validation of EMT signature.** (A) Internal validation (Kaplan-Meier curve, ROC curves) based on the TCGA test cohort (n = 316) with the same cut-off from training cohort. (B) External validation cohort (Kaplan-Meier curve, ROC curves) based on the GSE158309 cohort (n = 461) with the same method.

Next, the EMT signature was validated in the TCGA internal cohort using the same risk model and cut-off value from the training cohort. The testing cohort, which included 316 samples, was divided into high- and low-risk groups, showing significant survival differences (*P < 0.001*). The AUCs at 1-, 3-, and 5-year OS were 0.849, 0.739, and 0.725, respectively (Fig. 3A). Similarly, the GSE158309 external cohort, which included 461 samples, was divided into high-risk and low-risk groups, similar to the results of the TCGA testing cohort (*P < 0.001*); the AUCs at 1-, 3-, and 5-year OS were 0.672, 0.688, and 0.681, respectively (Fig. 3B).

To statistically validate the consistency of the model's discriminatory power, we performed DeLong's test for ROC comparison, the results revealed no significant difference in AUCs between the training cohort, the internal test cohort, and the GSE158309 external cohort at 1, 3, or 5 years (all *P > 0.05*), confirming the robust internal stability of the EMT signature.

### 3.3 Prognostic value for such model and comparison with several other independent models

**Fig. 4:**
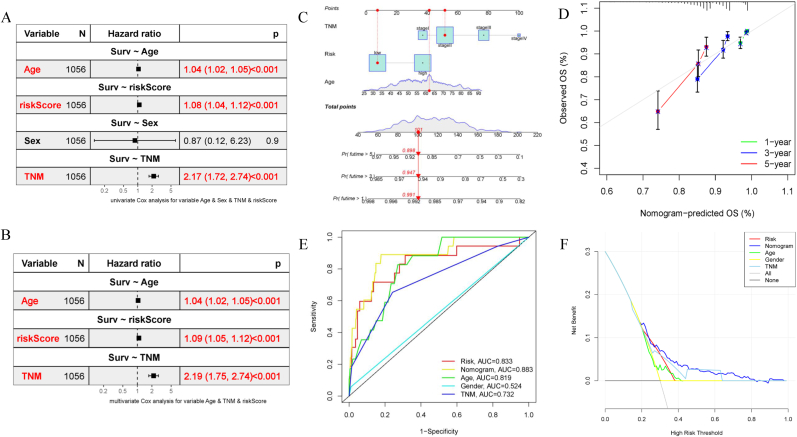
**Establishment and estimation of EMT signature.** Univariate (A) and multivariate (B) Cox regression analyses exhibited that the signature could serve as prognostic factors with the combination of clinical parameters (Age, TNM stage). (C) Prognostic model, including risk score, age, and TNM stage, was employed in predicting the survival rate at 1, 3, and 5-year. (D) Calibration curves at 1, 3, and 5-year between the prognostic and ideal models. (E) ROC curves compared the AUCs of different characters (such as risk, nomogram, Age, Gender, and TNM stage). (F) Decision curve analysis of all the parameters and the nomogram based on EMT signature performed the best value.

After an independent prognosis analysis (Fig. 4A–B), three independent prognostic factors (Age, TNM, and risk score) were selected to establish a nomogram for quantifying the prognostic value of the EMT signature, thereby assessing the survival rate at 1, 3, and 5-year. For instance, when the total number of points was 101, the 1-, 3-, and 5-year OS rates were 0.991, 0.947, 0.898 respectively (Fig. 4C), where a higher total points score indicated a greater risk of death and a poorer prognosis. The C-index used to evaluate the predictive ability of this signature was 0.719, indicating a medium accuracy. The calibration curve roughly fitted the ideal model regardless of deviation, indicating that the accuracy of the model was relatively high (Fig. 4D). We also visualized the AUCs of all the indicators (Fig. 4E). We found that the nomogram accurately predicted the survival rate (AUC = 0.883). Moreover, decision curve analysis was conducted to explore the best variable, and we observed that the nomogram was better than the other parameters, demonstrating distinct benefits (Fig. 4F).

**Fig. 5:**
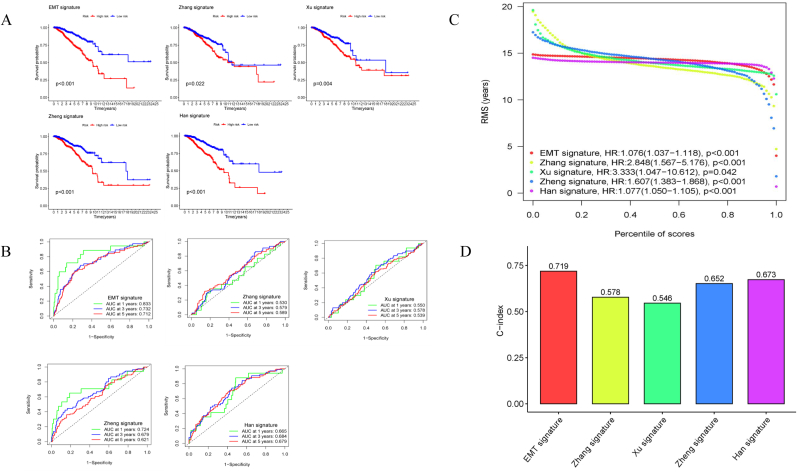
**Comparison of the prognostic value with other four models.** (A) Kaplan-Meier curves of EMT signature and other four models (such as Zhang signature, Xu signature, Zheng signature, and Han signature) with the same cut-off value. (B) AUCs at 1, 3, and 5-year were shown in ROC curves of the above-mentioned models. (C) Root mean square (RMS) of the respective percentile of risk scores in these five models. (D) Comparison of C-indexes in each model.

The EMT signature was further confirmed by comparison with the other four models associated with EMT or the immune genome [16]. Zhang et al. identified eight-gene signatures (*BIRC3*, *BTN3A1*, *CSF2RB*, *GIMAP7*, *GZMB*, *HCLS1*, *LCP2*, and *SELL*) [17], and Xu et al. constructed a five-gene signature (*CCR2*, *GPR183*, *CCR5*, *CCR4*, and *P2RY13*) [18] and a ten-gene model (*PFKL*, *SLC25A1*, *SERPINE1*, *TAPBPL*, *CXCL11*, *HLA-A*, *TCF7L2*, *TANK*, *IL12B*, and *IL18RAP*) was from Zheng's research [19]. An eight-genes signature was analyzed by Han's team (*ULBP2*, *ADRB1*, *TSLP*, *MIA*, *IL27*, *IFNE*, *SCG2*, and *NR0B1*) [20]. The survival and ROC curves at 1, 3, and 5-year were drawn using the same cut-off value (Fig. 5A–B). The high- and low-risk groups showed significant differences in OS (*P < 0.05*), and the AUCs for the EMT signature at 1, 3, and 5-year were the largest. The novel model exhibited a relatively high HR value (HR = 1.076) and the highest C-index (0.719), thereby proving the robustness of the EMT signature (Fig. 5C–D). Collectively, our EMT signature in the present work showed a more accurate predictive ability, seemingly possessing great potential value.

### 3.4 Gene function analysis and correlation analysis between clinical parameters

**Fig. 6:**
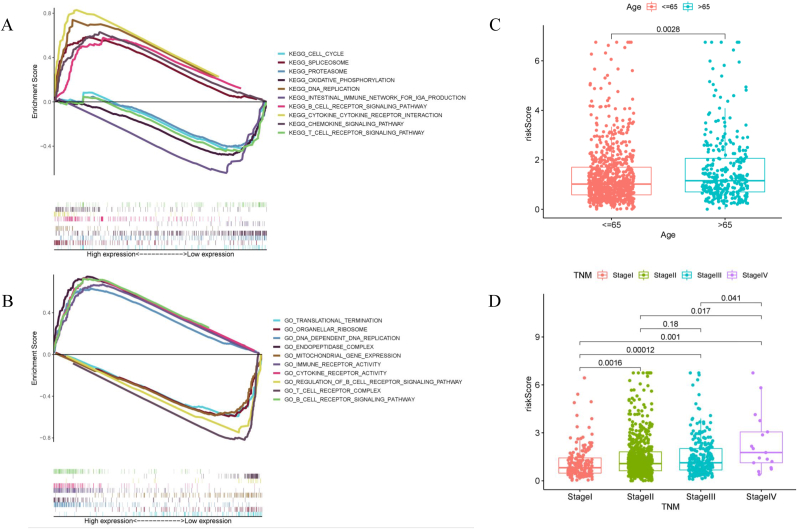
**Gene set enrichment analysis and correlation analyses.** (A) KEGG enrichment analysis. The top 5 enrichment pathways in high-risk and low-risk groups. (B) GO enrichment analysis. The top 5 enrichment pathways in high-risk and low-risk groups. Correlation between EMT signature and clinical parameters such as age (C) and TNM stage (D).

Based on the EMT signature, 1056 patients were divided into high-risk and low-risk groups for gene set enrichment analysis to explore the potential mechanisms. Gene enrichment analysis suggested that the low-risk group was significantly enriched in the immune pathways. KEGG analysis revealed the involvement of the B-cell receptor signaling pathway, cytokine-cytokine receptor interaction, intestinal immune network for production, T-cell receptor signaling pathway, and chemokine signaling pathway (Fig. 6A). GO analysis revealed the involvement of immune receptor activity, cytokine receptor activity, and the regulation of the B-cell receptor signaling pathway, T-cell receptor complex, and B-cell receptor signaling pathway (Fig. 6B). Additionally, the association between risk score and clinical variables from the nomogram (Age, TNM stage) was acquired by the “limma” package between high-risk and low-risk group. The risk score between the elderly group (>65 years) and the younger group (≤65 years) was different (Fig. 6C). In addition, there was differential expression in the risk scores between TNM stages, except for stages II and III (Fig. 6D).

### 3.5 Relationship between EMT signature and immune infiltration

**Fig. 7:**
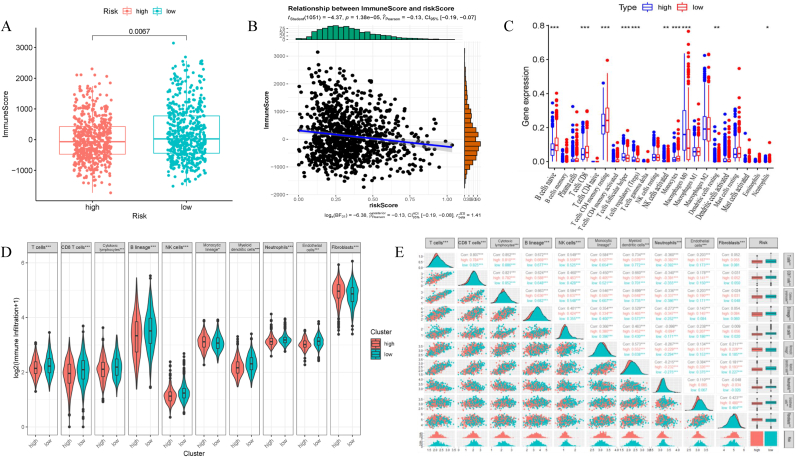
**Immune landscape in high-risk and low-risk groups.** (A) Wilcoxon test compared the differences between immune scores and EMT risk groups (*P < 0.05*). (B) Spearman's analysis revealed a negative correlation between immune scores and risk scores. (C) Multiboxplot revealed the expression difference of 22 immune cells between low-risk and high-risk groups with the method of CIBERSORT (Wilcoxon test, *∗∗∗P < 0.001, ∗∗P < 0.01, ∗P < 0.05*). (D) MCP-counter estimated 10 immune cells' abundance in two groups. (E) Spearman's analysis between the risk group and 10 immune cells.

In this step, we explored the differences in immune cell infiltration between high- and low-risk groups. First, we calculate the immune and ESTIMATE scores for each sample using the ESTIMATE algorithm. The low-risk group had a higher immune score (*P = 0.0067*) (Fig. 7A), and the risk score was negatively correlated with the immune score (*P < 0.001*, R = −0.13) (Fig. 7B), indicating a more active immune microenvironment in low-risk patients. Next, CIBERSORT was used to measure the relative infiltration proportion of 22 immune cells, and the infiltration differences between the high- and low-risk groups were compared (Fig. 7C). This analysis revealed that the low-risk group was notably enriched for effector immune cells such as CD8^+^ T cells and activated NK cells, which were associated with anti-tumor immunity, while the high-risk group showed a higher proportion of potentially immunosuppressive cells, including M2 macrophages and resting CD4^+^ memory T cells. In addition, we explored the infiltration of 10 primary immune cells and analyzed their correlation with the risk score to supplement the MCP-counter method. The results showed that most of these immune cells were differentially expressed in the low-risk group, except for the cytotoxic lymphocytes and fibroblasts (Fig. 7D). Moreover, a significant spearman correlation showed that the risk score negatively correlated with the abundance of immune effector cells, including T cells, CD8^+^ T cells, and myeloid dendritic cells, confirming the cellular composition findings and indicating an immune-effector-rich microenvironment in low-risk patients (Fig. 7E). Thus, the EMT signature stratified patients into subgroups with distinct immune cell infiltration patterns.

### 3.6 Mutational status and assessment of immunotherapy response

**Fig. 8:**
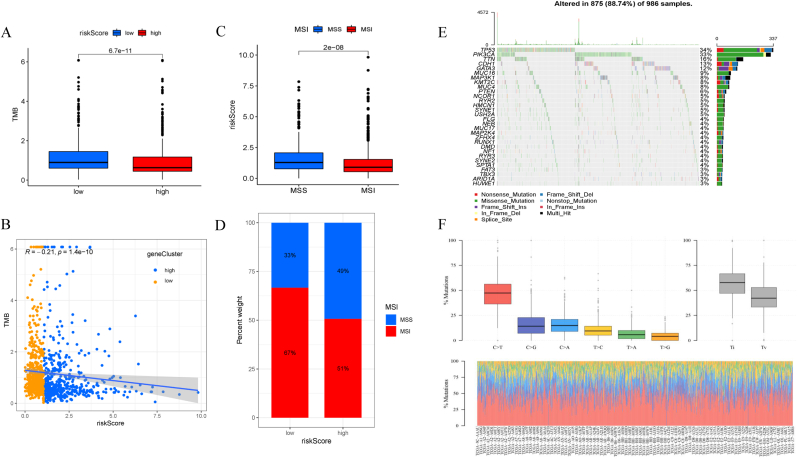
**Analysis of MSI and TMB.** (A) Low-risk group showed higher TMB (*P < 0.05*). (B) TMB was negatively correlated with EMT risk score (R = −0.21, *P < 0.05*). (C) Low-risk group exhibited high levels in MSI group when compared to that in microsatellite stability group (MSS). (D) Proportion of MSI and MSS in two risk groups. (E) Waterfall diagram of the genetic mutation status in the TCGA-BRCA cohort. (F) Detailed base pairs mutation in the TCGA-BRCA cohort.

As has been acknowledged that immune status was closely correlated to tumor mutation burden (TMB) as it could lead to anticancer immunity [21]. Therefore, the present study used the mutation data obtained from the TCGA-BRCA dataset to calculate the TMB for each sample and compare the differences between the high-risk and low-risk groups. The low-risk group had a higher TMB, and the risk score was negatively correlated with TMB (Fig. 8A–B), indicating a more sensitive response to EAG-targeted therapy via the suppression of immune pathways. Microsatellite instability (MSI) is defined as a DNA mismatch repair system disorder that can trigger extensive mutations known to cause abundant mutant neoantigens in cancer [22]. Previous studies suggested that patients with higher MSI would be more sensitive to immunotherapy [23,24]. Our results showed that the low-risk group had a higher MSI, demonstrating that patients could benefit more from immunotherapy (Fig. 8C–D). The waterfall chart presented 30 genes with the highest mutation rates, and the most common mutation types included missense mutations, nonsense mutations, and frameshift deletions (Fig. 8E). Transformation rate from cytosine to thymine is the highest, which was processed by the “matfools” R package (Fig. 8F), commonly attributed to spontaneous deamination [25] or APOBEC enzyme activity [26]. This mutational burden may associate with immunogenicity and immunotherapy sensitivity [27].

**Fig. 9:**
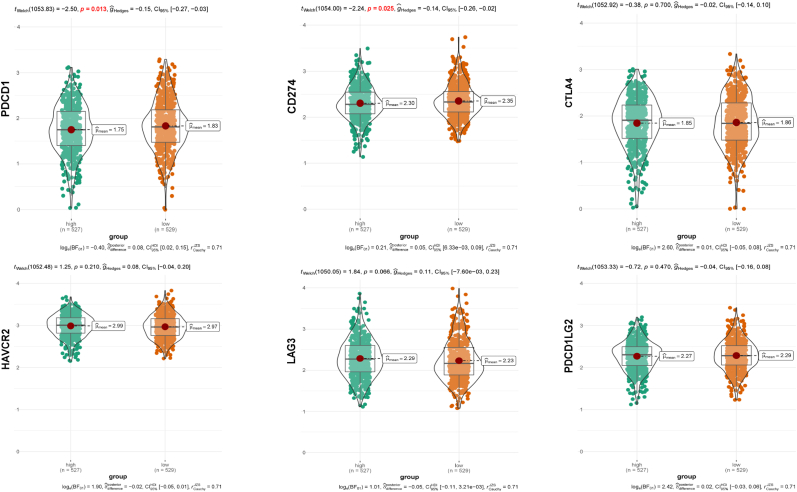
**Differential expression of ICIs between low-risk and high-risk groups classified by EMT signature.***PDCD1* and *CD274* showed higher expressions in low-risk group (*P < 0.05*).

**Fig. 10:**
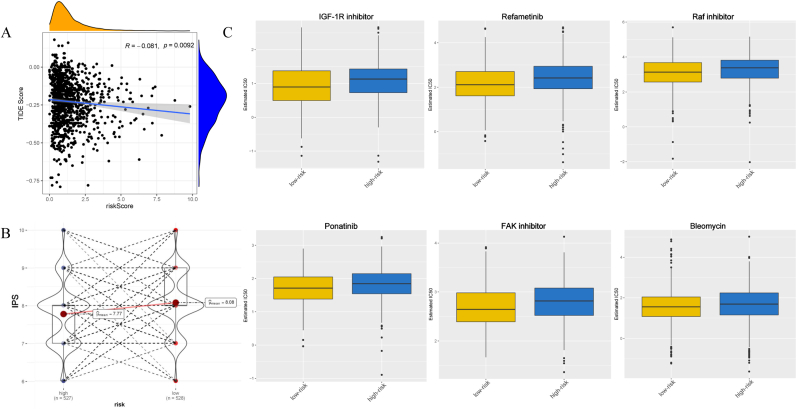
**Assessment of patients' sensitivity to immunotherapy and chemotherapy.** IC50 of 138 chemotherapeutic drugs was estimated by patients' baseline gene expression and drug sensitivity data in almost 700 cancer cell lines. (A) TIDE score was negatively correlated with EMT risk score (R = −0.081, *P < 0.05*). (B) IPS showed a higher level in low-risk group. (C) The top 6 chemotherapeutic drugs in low-risk group (*P < 0.05*).

The use of ICIs in immunotherapy has become a promising treatment option for recurrent and metaplastic BRCA. Our study explored the correlation between EMT signature and ICIs biomarkers, extrapolating the relationship between risk stratification and immunotherapy. *PDCD1*, *CD274*, *CTLA4*, *PDCD1LG2*, *LAG3*, and *HAVCR2* were selected for validation. Our results demonstrated statistically significant differential expression of *PDCD1* and *CD274* (*P < 0.05*). The expression levels of *PDCD1* (μmean = 1.83) and *CD274* (μmean = 2.35) were higher in the low-risk group (Fig. 9). We adopted the TIDE score and IPS to assess immunotherapy response, which showed that the risk score was negatively correlated with the TIDE score (*P = 0.0092*, R = −0.081) (Fig. 10A). Moreover, the low-risk group showed a higher IPS (Fig. 10B), indicating that immunotherapy was more beneficial for this group.

### 3.7 Association of such EMT signature with chemotherapy

The combination of chemotherapy and immunotherapy has been shown to exert better curative effects than individual therapies. In this study, the drug sensitivity analysis was based on computational predictions using the pRRophetic algorithm. These in silico results provide a prioritized list of candidate drugs for further investigation. We applied this approach to explore potential differences in drug response between the risk groups. pRRophetic is an R package that involves nearly 700 cell lines and 138 chemotherapeutic drugs and uses baseline gene expression and drug sensitivity data to predict clinical response in cancer cell lines [28]. We estimated the IC50 values of 138 drugs for each BRCA patient. The estimated IC50 values for the 50 drugs were statistically different between high risk and low risk groups (Wilcoxon test, *P < 0.05*) (Supplementary Table 2). Interestingly, the estimated IC50 values for 50 drugs were lower in the low-risk group. Six drugs had the highest estimated IC50 values (Fig. 10C). Our results suggest that patients at low risk were more sensitive to IGF-1R inhibitors such as Refametinib, Raf inhibitor, Ponatinib, FAK inhibitor, and bleomycin. These results indicated that the EMT signature could potentially guide the use of targeted drugs in clinical treatment.

### 3.8 Immunohistochemistry level of EMT signature genes

**Fig. 11:**
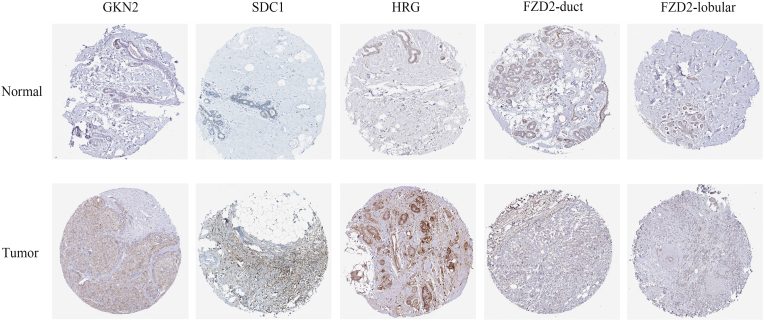
**Protein expressions of four meaningful EMT signature genes in normal breast and BRCA specimens from HPA database.***GKN2, SDC1, and HRG* proteins were not detected in normal breast tissues, but were low-medium stained in BRCA tissues. *FZD2* proteins were highly stained in normal breast myoepithelial cells, but not detected in glandular cells. Duct carcinoma tissues didn't show the staining of *FZD2* protein, lobular Carcinoma detected low-medium staining of *FZD2* protein.

To further explore the protein expression characteristics of these EMT signature genes, we used IHC images and staining intensity data from the HPA database (Fig. 11). Finally, four genes (*GKN2, SDC1, HRG, and FZD2*) were differentially expressed in the normal breast and BRCA tissues. In particular, *GKN2, SDC1*, and *HRG* showed low-medium staining in BRCA tissues, but not in normal tissues. *FZD2* was highly stained in myoepithelial cells but hardly stained in glandular cells of normal breast tissues. Correspondingly, opposite staining results were observed in duct carcinoma and lobular carcinoma; *FZD2* showed no staining in duct carcinoma but medium staining in lobular carcinoma. These results not only represent the expression of EMT signature genes in BRCA but also reveal the possibility that the same gene may have completely different molecular mechanisms with different pathological types in BRCA, which has great potential for guiding further research.

## 4 Discussion

EMT is the initial step in tumor migration and colonization [3]. EMT executes all molecular signaling activities at multiple levels. Alterations in the phenotype of epithelial and mesenchymal cells trigger a subsequent regulatory network [3,4]. Additionally, the process mainly transforms into the tumor microenvironment after activation by intercellular signals [29]. Accumulating evidence suggests that EMT can induce immune evasion to promote invasion and metastasis of cancer cells [30,31]. Moreover, the transitional process possesses excellent plasticity, which leads to dynamic intermediary phases, thereby enhancing the complexity of the exploration of EMT biomarkers. Therefore, determining the functional role of EMT in metastasis and identifying potential targets remain an area of active investigation.

Our signature was derived from a rigorous bioinformatics workflow, identifying key genes whose expression patterns stratify patients into distinct risk groups with validated prognostic value. Previous studies have revealed that several gene biomarkers can stratify high- and low-risk patients who could benefit from personalized therapy. However, the polymorphism of EMT, which includes a variety of upstream and downstream channels, limits its accuracy [32]. In the present study, we investigated the immunological branches of EMT. First, we identified a specific EAGs matrix containing 381 genes for subsequent analysis. Subsequently, a novel EMT signature was established after a series of statistical analyses including *GKN2*, *FZD2*, *NDRG2*, *SCUBE2*, *ALX4*, *CCL19*, *SDC1*, *EZR*, *CPEB1*, and *HRG* genes. The reliability of this signature was validated using the internal cohort obtained from the TCGA-BRCA dataset and external cohort of GSE158309. The prognostic independence of such signatures made it eligible for a nomogram that included clinical parameters (Age, TNM stage) to predict the survival rate at 1, 3, and 5-year. Subsequently, four previously established gene expression models with the same cut-off values were used for model comparisons. Our novel EMT signature is more effective in predicting the prognosis of patients with BRCA.

Functional enrichment analysis confirmed that the different risk groups are characterized by fundamentally different immune landscapes, providing a biological basis for the prognostic difference. KEGG and GO analyses showed that the immune infiltration pathways were enriched in the low-risk group. Thus, the present study focused on the immune pathways in the tumor microenvironment. Immune and stromal cells constitute the tumor microenvironment in BRCA, which plays a different role in tumor promotion and suppression. Cancer cells can reprogram stromal cells, whereas stromal lymphoid and myeloid cells can repress or promote carcinogenesis [33]. It has been reported that the density, composition, and arrangement of infiltrating immune cells are variable and associated with prognosis, immunotherapy, and chemotherapy response [34]. However, such immune infiltration information can be accessed using diverse computational deconvolution methods including ESTIMATE, CIBERSORT, and MCP-counter. These three algorithms confirmed a higher immune cell infiltration in the low-risk group. Our results not only suggested a close relationship between the EMT signature and immune infiltration but also attested to the heterogeneity of immune infiltration, which could explain the different survival rates between patient groups that were defined by the EMT signature.

Our model intentionally captured genes with dual roles in both EMT and immune regulation, indicating that it would identify an EMT subtype intrinsically linked to immune microenvironment reprogramming. For instance, *FZD2*, a trigger of the canonical Wnt/β-catenin EMT pathway, had also been implicated in promoting M2-like polarization of macrophages in nasopharyngeal carcinoma [35]. As the predominant cell surface proteoglycan in epithelial tissues, *SDC1* played a well-established role in driving carcinoma progression and facilitating EMT [36]. It had been also acknowledged as a key immunity-related gene upregulated in triple-negative breast cancer (TNBC). High tumor *SDC1* coupled with low cancer associated fibroblasts (CAFs) correlated with poor prognosis and reduced tumor-infiltrating lymphocytes (TILs), indicating its role in driving TNBC cell migration via TGFβ1/Smad and EMT [37]. Notably, *HRG* could regulate macrophage polarization and angiogenesis, potentially serving as a pleiotropic node connecting EMT, immune response, and stromal reprogramming [38]. The co-inclusion of these functionally ambivalent genes supported the premise that our EMT signature may delineate a specific, immunologically active EMT trajectory in breast cancer, extending its relevance beyond pure prognosis to potential therapeutic stratification.

Further analysis linked the low-risk, immune-rich phenotype to genomic features and biomarkers associated with favorable responses to immunotherapy. Previous studies may provide outstanding support for patients sensitive to immunotherapy and guide clinical management [39,40]. We evaluated the association between the EMT signature and several putative immunotherapy biomarkers, such as TMB, MSI, and ICIs. High TMB, MSI, and ICIs levels are usually considered predictive biomarkers of patient responses to immunotherapy. Our results suggested that the low-risk group exhibited higher TMB and MSI levels and higher expression of *PDCD1* and *CD274*, suggesting a sensitive immunotherapy response. Next, the 30 most mutated genes and the mutational situation of the base pairs in somatic BRCA cancer cells were determined, which would further provide directions for experimental design. Moreover, our study adopted the TIDE score and IPS to assess the sensitivity to immunotherapy in different groups. The results showed that the TIDE score was negatively correlated with the risk score, and that the IPS was higher in the low-risk group. Thus, the aforementioned results provide robust evidence that patients with low-risk EMT signatures can benefit from immunotherapy.

Beyond immunotherapy, the EMT signature also showed potential in predicting differential sensitivity to conventional and targeted chemotherapeutic agents. The pRRophetic analysis revealed that patients with low risk exhibited a lower estimated IC50 for most drugs, indicating that the low-risk group was more sensitive to chemotherapy. These drugs including vinorelbine and gemcitabine are widely used in clinical practice. Overall, this novel EMT signature may be a useful tool to assist clinicians in prognosis prediction and may be used to guide personalized clinical therapies (including targeted therapy, immunotherapy, and chemotherapy).

Despite its excellent predictive ability to identify high- or low-risk BRCA, predict patient prognosis, and individualize cancer treatment, our study had several limitations. First, the conclusions were derived solely from bioinformatic analysis and lack of experimental validation (in vitro and in vivo) of the signature genes’ functions and their mechanistic roles in the EMT-immune axis. Second, while validated in TCGA and GEO datasets, the predictive performance of the signature, particularly for immunotherapy response, had not been assessed in a dedicated prospective clinical cohort of patients receiving uniform immunotherapy. Thus, prospective analysis is required to explore the correlation between EMT signatures and immunotherapy. Furthermore, detailed molecular mechanisms underlying the EMT immune pathway remain unclear. Therefore, clarifying the mechanism of EAGs in the EMT-immune pathway and identifying precise targets that simultaneously act on EMT and immune infiltration seems worthwhile.

In conclusion, we constructed and validated a novel 10-gene EMT signature that was robustly associated with the tumor immune microenvironment in this study. This signature not only accurately predicted the prognosis of breast cancer patients but also effectively distinguished subgroups with divergent immune infiltration patterns, tumor mutational burden, and potential responses to immunotherapy and chemotherapy. It thus provided a new and promising molecular tool for prognostic stratification and personalized treatment strategies, particularly in the selection of immunotherapy and chemotherapy. Future experimental validation and prospective clinical studies would be crucial to translating this signature into clinical practice.

## Consent to participate

N/A.

## Consent to publish

N/A.

## Author contributions

All authors contributed to the data analysis, drafting, and revision of the article, approved the final version to be published, and agreed to be accountable for all aspects of the work.

## Ethics approval

Ethics approval is not required.

## Funding

This work was kindly supported by grants from the Changzhou Medical Center (CMCB202401), the Jiangsu Provincial Health Commission General Project (MQ2024036), the Excellent Post-doctoral Program of Jiangsu Province (2022ZB820), the Top Talent of Changzhou ''The 14th Five-Year Plan'' High-Level Health Talents Training Project (2022CZBJ065), and the Post-doctoral Foundation of China (2022M720543).

## Declaration of competing interest

The authors declare that they have no known competing financial interests or personal relationships that could have appeared to influence the work reported in this paper.

## Data Availability

Research data supporting this publication are included in the paper and its supplementary files. RNA-Seq data, and clinical parameters of breast cancer samples were downloaded from The Cancer Genome Atlas (TCGA) (https://portal.gdc.cancer.gov/) and the Gene Expression Omnibus (GEO) database (https://www.ncbi.nlm.nih.gov/geo/). The use of this data complies with the TCGA and GEO Data Use Policy and the terms specified in the Data Use Certification Agreement. The detailed datasets analyzed during the current study are available from the corresponding author upon reasonable request.

## Appendix A Supplementary data

Supplementary Figure 1. **Recognition of the differentially expressed EAGs**. (A) Heatmap showing the expression of 8130 differentially expressed genes. (B) Venn diagram showing the interaction between TCGA-diff genes and EAGs.Multimedia component 1

Supplementary Table 1. The 31 genes associated with OS.Multimedia component 2

Supplementary Table 2. Estimated IC50 of 50 drugs which were illustrated to be different in two groups.Multimedia component 3
