# Supplementary figures and images for: Recognition of immunogenomic signature and prognostic value of the subtype of epithelial-mesenchymal transition in breast cancer
Source: Biochem Biophys Rep. 2026 Jan 19;45:102456. doi: 10.1016/j.bbrep.2026.102456 (PMC12857188; doi:10.1016/j.bbrep.2026.102456)

A

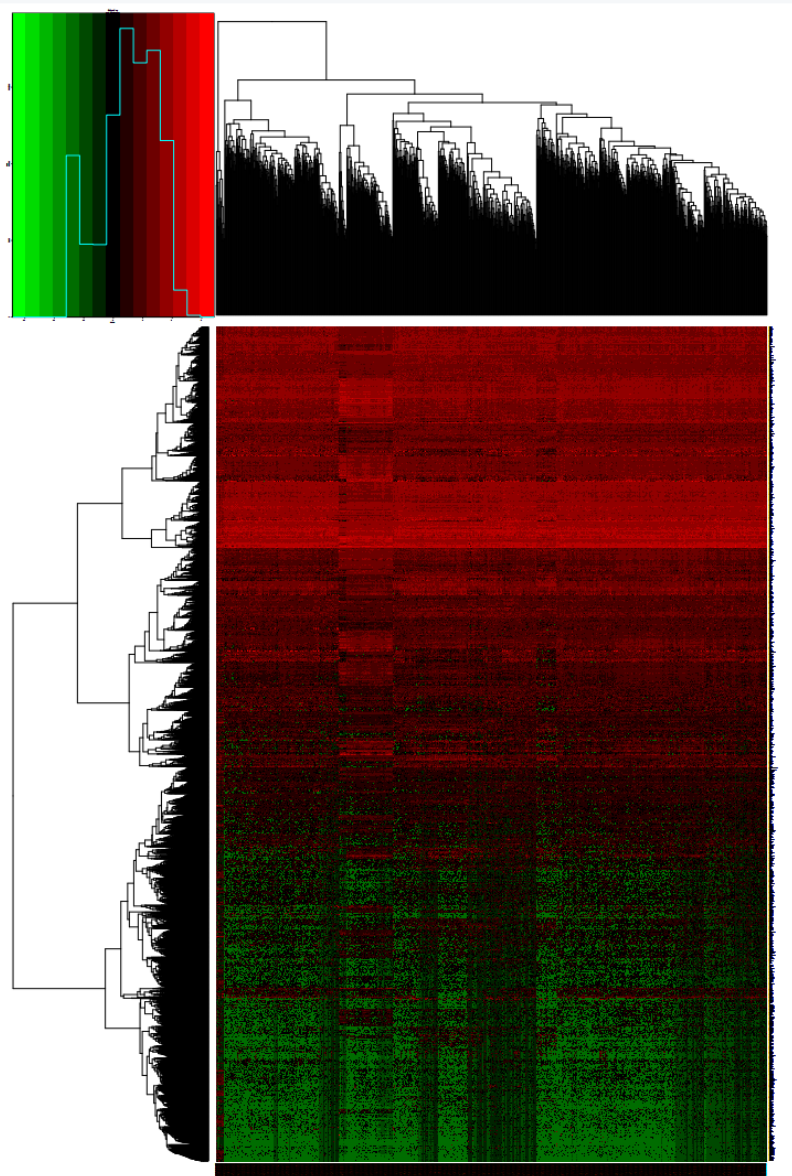

B

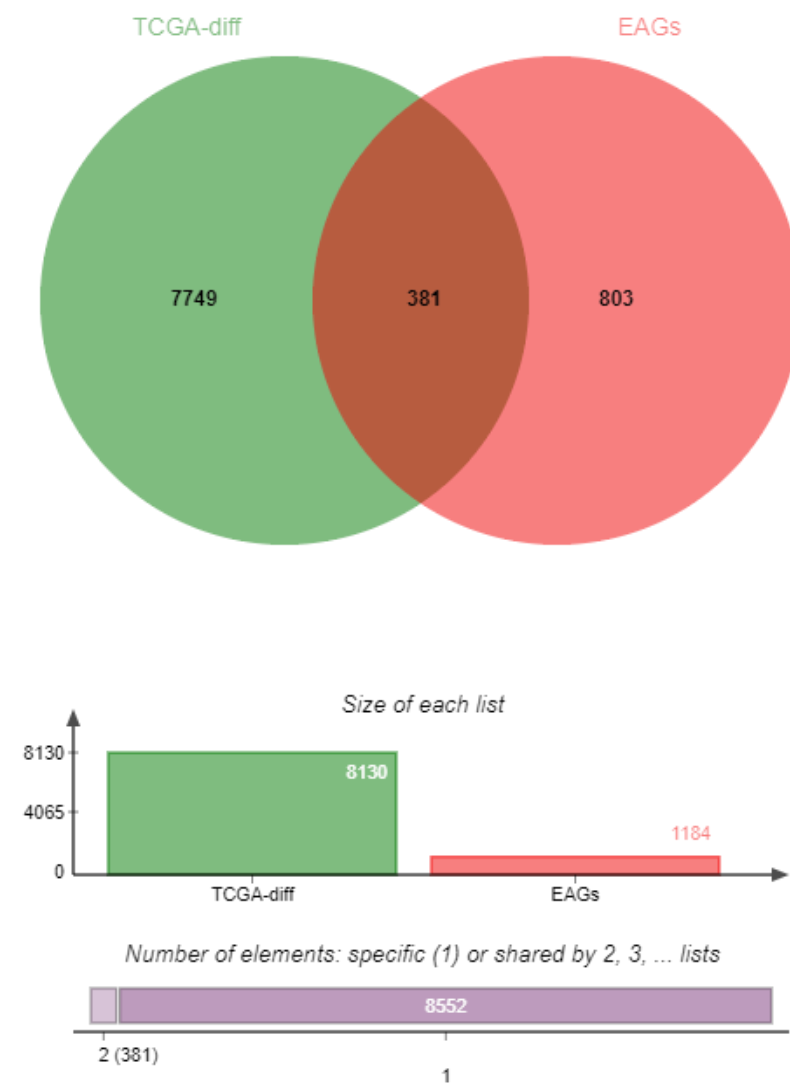

Supplement: Supplementary file 1 — Supplementary Figure 1. Recognition of the differentially expressed EAGs. (A) Heatmap showing the expression of 8130 differentially expressed genes. (B) Venn diagram showing the interaction between TCGA-diff genes and EAGs.Multimedia component 1 [file mmc1.pdf]
